# Zinc-solubilizing bacterial consortia: a promising approach for zinc biofortification of crops

**DOI:** 10.3389/fmicb.2025.1575514

**Published:** 2025-06-25

**Authors:** Viabhav Kumar Upadhayay, Saurabh Gangola, Gohar Taj, Kumar Gaurav, Anju Rani, Sunil Kumar, Shivanshu Garg, Gaurav Gupta, Haider Ali, Sazada Siddiqui, Saad A. M. Alamri, Amit Mittal, Sulaiman A. Alrumman, Mayank Pandey

**Affiliations:** ^1^Department of Microbiology, College of Basic Sciences & Humanities, Dr. Rajendra Prasad Central Agricultural University, Pusa, Samastipur, Bihar, India; ^2^Department of Microbiology, Graphic Era Deemed to be University, Dehradun, India; ^3^Department of Molecular Biology & Genetic Engineering, College of Basic Sciences and Humanities, GB Pant University of Agriculture and Technology, Pantnagar (U. S. Nagar), Uttarakhand, India; ^4^School of Agriculture, Graphic Era Hill University, Dehradun, India; ^5^Department of Biochemistry, College of Basic Sciences and Humanities GB Pant University of Agriculture and Technology, Pantnagar (U. S. Nagar), Uttarakhand, India; ^6^Centre for Research Impact & Outcome, Chitkara College of Pharmacy, Chitkara University, Punjab, India; ^7^Center for Global Health Research, Saveetha Medical College, Saveetha Institute of Medical and Technical Sciences, Saveetha University, Chennai, India; ^8^Department of Biology, College of Science, King Khalid University, Abha, Saudi Arabia; ^9^Department of Allied Sciences, Graphic Era Hill University, Bhimtal, India; ^10^Department of Biotechnology, Kumaun University, Nainital, India

**Keywords:** bacteria, biofortification, consortium, plant nutrient, zinc

## Abstract

The term “zinc-solubilizing bacteria” (ZSB) refers to a specific group of soil bacteria that are associated with zinc-solubilizing activity in the soil through a variety of mechanisms. The functional use of ZSB has been proposed for the zinc (Zn) biofortification of crops to address Zn malnutrition. The application of zinc-solubilizing bacterial inoculants that harbor significant plant probiotic traits offers an eco-friendly approach to producing crops with improved Zn content in various edible parts of plants. In soil, ZSB solubilize complex forms of Zn compounds by producing organic acids and employing other mechanisms (such as the secretion of “siderophore,” extrusion of “proton,” expression of “oxidoreductive systems” on cell membranes, and secretion of “chelated ligands”), making the resulting soluble form of zinc readily accessible to plants. ZSB also act as plant growth stimulators, demonstrating both direct and indirect mechanisms that promote robust plant growth. In recent years, the application of two or more ZSB strains in a consortium has gained attention as a cost-effective alternative for Zn biofortification. This approach may serve as a promising strategy for promoting plant growth and optimizing yield performance. This review discusses various methods of Zn biofortification, highlighting ZSB and their consortia in increasing Zn content in grains and other edible crop parts, as well as the mechanisms involved in Zn solubilization by these bacteria. This insight paves the way for developing eco-friendly strategies that integrate microbial-based solutions to improve crop nutrient bioavailability.

## 1 Introduction

The interest in producing a “biofortified crop enriched with zinc (Zn)” has increased due to the prevalence of Zn malnutrition. A large proportion of the world's population suffers from zinc malnutrition due to the consumption of crop-based food with a low content of this important micronutrient (Kiran et al., 2022; Lowe et al., 2024; Stiles et al., 2024). Zn is essential for all living entities (plants, animals, humans, and microorganisms; Srivastava et al., 2025). The lack of Zn drastically affects both plants and humans (Younas et al., 2023). Several ailments in humans are associated with Zn deficiency, which is prevalent in developing nations where people rely on food crops and do not take other health supplements to fulfill their need for essential micronutrients (Hussain et al., 2018; Wani et al., 2017; Khan A. et al., 2022). The concept of biofortification is a significant contribution from the global scientific communities involved in agricultural research. A number of strategies are employed to enhance the micronutrient concentration in edible crops (Avnee et al., 2023; Upadhayay et al., 2018, 2019). These strategies may include agronomic methods, plant breeding, and transgenic approaches to combat micronutrient deficiency (Garg et al., 2018; Naik et al., 2024; Upadhayay et al., 2022a,b,c). However, these approaches can be costly and challenging to implement in areas where underprivileged populations reside. There is a current demand for inexpensive biofortification methods; therefore, the use of ZSB as bioinoculants is a promising tactic for Zn biofortification and plant growth (Khan et al., 2019; Kumar A. et al., 2019; Hussain et al., 2018; Rahman et al., 2024). Soil microorganisms, especially agriculturally relevant taxa, play an imperative role in plant growth by exhibiting multiple “plant growth-promoting traits.” These traits include biological nitrogen fixation, mineral solubilization, and the biosynthesis of phytohormones, siderophores, exopolysaccharides (EPS), and hydrogen cyanide (HCN; Khoso et al., 2024). Additionally, these microorganisms enhance the bioavailability of essential nutrients such as zinc (Zn), phosphorus (P), and potassium (K) by solubilizing their complex forms in the rhizospheric microenvironment (Li et al., 2021). This process improves nutrient absorption by plants, promoting their growth and overall health. ZSB are either rhizospheric or endophytic microorganisms that catalyze the solubilization of insoluble Zn compounds. The production of organic acids (particularly gluconic acids) and the secretion of chelating components (specifically siderophores) by ZSB are key mechanisms for Zn solubilization (Singh D. et al., 2024; Mishra et al., 2025; Sethi et al., 2025). Several ZSB have been studied to demonstrate their efficiency in the biofortification of plants with essential micronutrients, especially Zn (Bhatt and Maheshwari, 2020; Mumtaz et al., 2022; Upadhayay et al., 2022a,b,c; Pathak et al., 2024; Shakeel et al., 2024; Singh et al., 2025). Moreover, intensive application of chemical fertilizers shows a negative impact on the environment and disrupts soil health (Upadhayay et al., 2023), therefore, ZSB as potential bioinoculants could be used in agriculture for effective growth of crops (Hussain et al., 2018; Kamran et al., 2017). ZSB are excellent biostimulants as they contain multiple traits collectively determined as “plant growth-promoting traits” (Mumtaz et al., 2017). The traits include “phosphate solubilization” (Ali et al., 2023a), “potassium solubilization,” “nitrogen (N2) fixation,” “synthesis of phytohormones like indole-3-acetic acid (IAA)” (Othman et al., 2022), “1-aminocyclopropane-1-carboxylate (ACC) deaminase” (Sukhwal et al., 2023), “siderophores” (Costerousse et al., 2017; Ramesh et al., 2014; Upadhayay et al., 2024). The development of ZSB-based biofertilizers represents a sustainable and efficient alternative to conventional chemical fertilizers, offering comparable efficacy with reduced environmental liabilities (Sindhu et al., 2019; Upadhayay et al., 2018). Research into exploring “microbial consortia” as biostimulants is increasing worldwide. Compared to single strains, consortia exhibit greater potential for plant growth-promoting properties. Consortia, as “potential plant probiotics,” improve the growth, yield, and nutritional status of plants (Menéndez and Paço, 2020). However, exploring a consortium of ZSB could provide additional Zn biofortification benefits for several crops. There are only a limited number of studies available deciphering consortia-mediated zinc enhancement in crops (Ali et al., 2023a; Kasno et al., 2024; Singh et al., 2025), where increased accumulation of Zn in edible parts (such as grains and fruits,) constitutes a “biofortification event” (Upadhayay et al., 2022a,b). Thus, an increased level of Zn and other essential micronutrients enhances the nutritive value of food crops, which is an important step toward addressing micronutrient deficiencies in populations that depend on food crops as an essential component of their diet (Khan et al., 2019; Upadhayay et al., 2018, 2022a, 2024). The excessive application of chemical fertilizers is associated with soil health disruption and negative environmental impacts. Eventually, the use of ZSB-based consortia provides an alternative solution for sustainable agriculture by reducing reliance on agrochemicals, thus helping to maintain soil health with negligible environmental risks. This review article provides a concise overview of the biofortification benefits of ZSB and their consortia, as well as their effectiveness as biostimulants for enhancing crop growth.

## 2 Importance of zinc

Zn is an essential micronutrient required in minute amounts by all living organisms, including plants, prokaryotes, humans, and other animals (Khan et al., 2019; Hamzah Saleem et al., 2022; Stiles et al., 2024). Zn exhibits biological significance and has numerous public health implications (Wani et al., 2017) and is also considered the most abundant “transition metal” in organisms (Hussain et al., 2018; Wani et al., 2017; Daccak et al., 2022). Zn acts as a “core item” for the activation of several enzymes (Jin et al., 2024). It also serves a structural role in transcription factors (Wani et al., 2017) and is associated with other functions, including gene expression regulation (Zeng et al., 2021). A deficiency of Zn in humans may lead to the progression of several ailments. Approximately two billion people in developing nations are estimated to suffer from Zn deficiency (Martínez-Ríos et al., 2024). Symptoms such as delayed puberty, diarrhea, nail dystrophy, hyperammonemia, growth retardation, hypogonadism, erectile dysfunction, severe immune dysfunction or weakened immunity, alopecia, recurring infections, neurosensory disorders, and glossitis are associated with a lack of zinc (Hawrysz and Wozniacka, 2023; Sethi et al., 2025; Upadhayay et al., 2018, 2019, 2022a,b,c). The lack of this micronutrient can result in (a) impaired wound healing (Khan et al., 2019), (b) viral infections (such as HIV and HCV), (c) impaired spermatogenesis, (d) photophobia, (e) smell and taste impairment, and (f) loss of appetite (Hawrysz and Wozniacka, 2023; Pourmoradian et al., 2024). Zn also exhibits antioxidant activities and may protect against oxidative stress (Sethi et al., 2025). In addition to humans and animals, Zn plays multiple roles in several plant metabolic functions. A large number of enzymes, including carbonic anhydrase, RNA polymerases, alcohol dehydrogenase, and superoxide dismutase, require Zn for their activation (Khan S. T. et al., 2022; Natasha et al., 2022). Moreover, Zn plays a crucial role in protein synthesis. It is also indispensable for the metabolism of carbohydrates, nucleic acids, and lipids, contributing to a variety of biochemical and cellular processes (Hamzah Saleem et al., 2022). Zn deficiency in plants results in impaired enzymatic activity and inhibition of photosynthesis due to the reduction in the activity of enzymes associated with this process (Upadhayay et al., 2022a,b). Chlorosis of leaves, stunted growth, spikelet sterility, and increased susceptibility to injury (caused by high light intensity and temperature) and infection (from some fungal diseases) are also associated with severe Zn deficiency (Ali et al., 2023a; Bastakoti, 2023).

## 3 Concept of biofortification

**Figure 1:**
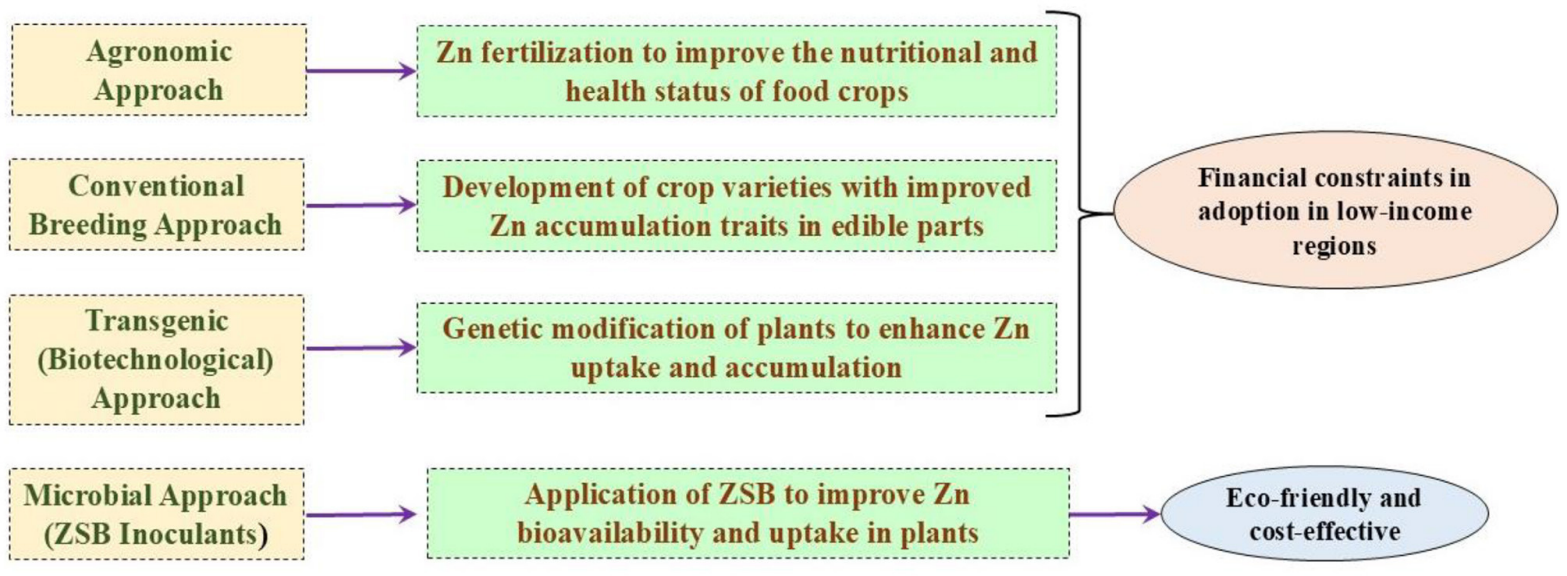
Representation of various approaches for Zn biofortification.

There is a current need to produce biofortified crops to feed a significant portion of the population suffering from Zn malnutrition. The lack of adequate micronutrient intake leads to “hidden hunger” (Upadhayay et al., 2018). Soils in various countries, including India, Turkey, Iran, Pakistan, and China, are deficient in micronutrients such as zinc (Khan A. et al., 2022; Upadhayay et al., 2022a). As a result, crops grown in these regions may also contain lower levels of Zn in their edible portions. A large segment of the population relying on such crops consequently faces Zn malnutrition if they lack access to health supplements containing sufficient Zn. The low phyto-availability of Zn in agricultural soils, due to its limited soluble fraction, adversely affects crop productivity and leads to nutritionally inadequate Zn content in the edible portions of food crops (Sethi et al., 2025). Therefore, the concept of “biofortification” is a viable approach to achieving adequate levels of Zn in plant edibles. This approach focuses on staple crops such as rice, wheat, maize, beans, and potatoes, using both conventional and modern approaches to increase nutritional value (Garg et al., 2018). Four important tactics—”dietary modification or diversification,” “supplementation,” “fortification,” and “bio-fortification”—are crucial to combat Zn deficiency, with the choice of implementing each tactic relying on several factors: (a) technical feasibility, (b) target group, (c) availability of resources, and (d) social acceptance (Ofori et al., 2022). Augmenting the micronutrient levels (especially “Zn” and “Fe”) in the crop's edibles (particularly “grain” and “fruit”) is referred to as “biofortification.” It is an effective tactic in modern agriculture for providing access to more nutritious and biofortified food to a large portion of the human population with limited resources (Riaz et al., 2020). The strategy of biofortification offers advantages by benefiting low-income households through the consistent supply of staple foods. Additionally, it gives a potential means of delivering naturally or biofortified foods to malnourished populations (Nestel et al., 2006). Some biofortified crops, including cereals, vegetables, legumes, and fruits, are providing micronutrients at required concentrations to targeted populations (Garg et al., 2018). The approaches to biofortification, including agronomic, breeding, and transgenics, are often costlier, labor-intensive, and slow (Upadhayay et al., 2022a). On the contrary, using ZSB presents an inexpensive and environmentally friendly approach to achieve biofortification benefits (Kushwaha et al., 2021; Hussain et al., 2018). Table 1 provides a comprehensive list of Zn-biofortified crop varieties (wheat, rice, maize, and sorghum), along with their respective countries of origin, as documented by HarvestPlus (https://bcr.harvestplus.org/varieties_released_list). Various biofortification strategies are illustrated in Figure 1. The biofortification of food crops can be achieved by adopting certain approaches.

**Table 1:** List of zinc-biofortified crop varieties released in different countries.

**Crop**	**Variety name**	**Country**
Wheat	MACS 4058, MACS 4028 (DURUM), HUW 711, HPBW01, HI 8777 (DURUM), HI 1633, DBW 332, BHU-5, BHU-31, BHU-3, BHU-25, BHU-1, PBW Zinc 2, Zn-Shakti, WB-02, PBW 771, PBW 757	India
	INIAF Okinawa	Plurinational State of Bolivia
	BRS 331	Brazil
	Borlaug100, Himgange, Zinc wheat-3, Zinc Gahun-2, Zinc Gahun-1, Panchakoshi	Nepal
	Akhbar-2019, Zincol-2016, TARNAB-REHBAR, TARNAB-GANDHUM-I, Nawab-21	Pakistan
	Nohely F2018	Mexico
	BARI-Gom33	Bangladesh
Rice	DRR Dhan 49, IET 28694	India
	Binadhan 20, BRRI dhan100, BRRI dhan102, BRRI Dhan62, BRRI Dhan64, BRRI Dhan72, BRRI Dhan74, BRRI Dhan84, BU Aromatic Dhan-2, BU Aromatic Hybrid Dhan-1	Bangladesh
	INPARI IR Nutri Zinc, INPARA 11, INPARA 12, Inpara 13 Fortiz	Indonesia
	IR120687-B-60-1-2-B, IR124029-B-13-1-1-B	Burundi
	INTA Las Minas	Nicaragua
	Fedearroz BIOZn 035	Colombia
	CIAT BIO-44 + Zinc	Plurinational State of Bolivia
	CENTA A-Nutremas	El Salvador
Maize	SGBIOH6, SGBIOH2, BIO-MZn01	Colombia
	INTA-Nutremas, Fortinica	Nicaragua
	ICTA HB-18ACP + Zn, ICTA B-15ACP + Zn, Fortaleza 17	Guatemala
	DICTA B03, DICTA B02	Honduras
	CENTA Porrillo 2020	El Salvador
Sorghum	Parbhani Shakti	India

### 3.1 Agronomic methods

It requires the application of zinc fertilizer to enhance the nutritional value of crops and increase their productivity. This strategic intervention not only provides vital Zn nutrients to plants but also serves as an effective and convenient solution for mitigating zinc deficiency. The intake of food derived from biofortified crops (enriched with Zn) improves the Zn-deficient state of consumers and improves the health status of individuals relying on such crop-based foods (Gomes et al., 2023). The most commonly used zinc fertilizer is zinc sulfate, which is an effective means to increase the Zn content in the edible parts of plants. It also improves both the growth and yield of plants (Tayyari et al., 2024). The application of “Zn,” either through soil or foliar spray, significantly enhances crop vigor and provides Zn biofortification of food crops (Hussain et al., 2018; Tayyari et al., 2024).

### 3.2 Plant breeding

The concept of breeding in biofortification aims to produce new varieties of crop plants with desirable traits related to optimal nutrient content and agronomic features (Naik et al., 2024). Plants generated from crosses between parent and recipient lines may possess the necessary characteristics (such as an improved Zn uptake mechanism in various parts of the plant, especially the edible portion; Xu et al., 2024). The primary goal of this strategy is to develop new varieties of crop plants with enhanced efficiency of Zn uptake and accumulation in edible parts (Garg et al., 2018). Numerous genetic studies have been conducted to increase Zn levels in edible (grain) parts. Candidate genes responsible for their involvement in the uptake and accumulation of iron and zinc in rice have been identified. Moreover, such genes are effectively employed in producing transgenic lines with traits for substantial accumulation/uptake of Zn and Fe (Swamy et al., 2016).

### 3.3 Transgenic approach

The modern biotechnology approach shows a promising role in the production of biofortified crops with desirable attributes of nutritional fortification in a very stable way. A number of transgenic crops (rice, cassava, oilseeds, and potatoes) have been developed with increased contents of Zn, Fe, vitamin A, and some essential amino acids (Hefferon, 2019). The role of the transgenic approach is numerous, and its current contribution to providing “biofortification benefits” to plants is outstanding. As a “realistic approach,” it can enrich a food-based crop plant with a particular type of micronutrient (a micronutrient that does not naturally exist in plants; Garg et al., 2018; Pérez-Massot et al., 2013). Developing crops through a transgenic approach shows improved Zn concentration in grains (Hefferon, 2019). Modulating the expression of transporters (especially “plant metal transporter proteins”) is the primary target of transgenics. Altered expression of transporters boosts the accumulation of Zn and Fe by plants (Krithika and Balachandar, 2016; Kumar A. et al., 2019; Krishna et al., 2023). Moreover, reducing the level of “phytic acid” in the grain is also important, as this organic component acts as an “anti-nutritional factor” (Kumar A. et al., 2019). Transgenics have also addressed this issue by lowering the concentration of such anti-nutritional compounds in food products (Kumar A. et al., 2019; Hefferon, 2019). The expressed transporter proteins utilize several metals (iron, zinc, cadmium, etc.). These metals travel from the soil to the root sections by acting as substrates for transporter proteins (Kumar A. et al., 2019). The overexpression of genes associated with specific transporter proteins (especially root transporter proteins) can improve the issue of lower Zn uptake from the soil to the plant (Roy et al., 2022). For instance, the expression of “*OsZIP9*,” a ZIP family transporter, was found to be crucial for Zn uptake in rice; it was expressed in the epidermal and exodermal cells of lateral roots, where its expression enhanced Zn accumulation under zinc-deficient conditions (Yang et al., 2020). Studies have shown that transgenic rice developed for increased levels of iron can address micronutrient (both Fe and Zn) deficiency (Majumder et al., 2022). One Fe transporter gene, namely, “*MxIRT1*,” is well-studied in apples and has applications in developing transgenic crops. Transgenic rice expressing this gene was found to achieve a three-fold increase in “Fe” and “Zn” (Tan et al., 2015). Typically, a significant portion of nutrients (Fe and Zn) is lost during the milling process (Hefferon, 2019). Therefore, a variety of rice (“transgenic high-yielding indica rice cultivar”) harboring the “ferritin gene” from another plant source (soybean) was developed to mitigate this issue (Paul et al., 2014). This particular cultivar demonstrated a “2.54-fold” increase in “Fe” concentration and a “1.54-fold” enhancement in “Zn” concentration. Two genes, namely, “*SferH-1*” (soybean ferritin) and “*OsNAS2*” (rice nicotianamine synthase), expressed in transgenic rice plants resulted in a considerable enrichment of the endosperm with two very important micronutrients (zinc and iron; Trijatmiko et al., 2016).

### 3.4 Zinc-solubilizing bacterial approach

Utilizing zinc-solubilizing bacterial inoculants to improve Zn micronutrient status provides a cost-effective strategy for biofortification (Upadhayay et al., 2018, 2022a,b,c). This environmentally friendly approach offers a sustainable solution for enhancing Zn levels in crop edibles (Hussain et al., 2018). ZSB inoculants solubilize insoluble Zn compounds through various mechanisms (Khan et al., 2019). These mechanisms may include (a) the secretion of “organic acids” by ZSB, and (b) the production of chelating molecules, especially “siderophores” (Bhatt and Maheshwari, 2020; Kumar S. et al., 2019). Microbes such as “rhizospheric microorganisms” and “endophytes” having Zn-solubilizing potential and other massive plant growth-elevating traits potentially increased the Zn concentration in staple crops (Costerousse et al., 2017; Mumtaz et al., 2020; Upadhayay et al., 2022a).

## 4 Zinc-solubilizing bacteria (ZSB): a green strategy for Zn biofortification

It is a well-known fact that Zn malnutrition affects a significant portion of the population in developing countries. Several ailments have been reported that occur due to Zn deficiency. The biofortification strategy is adopted to address the issue of Zn malnutrition by developing crops with high Zn density in edible portions. It is important to develop biofortified food crops, including “wheat,” “rice,” and “maize.” A large segment of the population depends on these staple crops for their basic diet. Fortified crops could be an effective means to curb Zn malnutrition. To develop biofortified crops with improved nutritional profiles, researchers are not only applying cutting-edge strategies but also actively exploring innovative approaches. However, most strategies are more expensive and may not be suitable for developing countries where the rural population predominates (Upadhayay et al., 2018, 2019). Moreover, the methods used for biofortification and food fortification do not always yield desirable results (Khan et al., 2019). Long-term application of fertilizers causes several issues, such as (a) deterioration of soil fertility, (b) disturbance of soil ecology, and (c) changes in the soil microbiome (Khan et al., 2023, 2024; Upadhayay et al., 2018, 2019). Therefore, alternative strategies are needed to deliver enhanced levels of Zn in edible portions of crops in a cost-effective manner. Thus, the application of ZSB presents an effective and green approach for Zn biofortification. ZSB are effective biostimulants that improve nutrient uptake by plants and exhibit a wide array of plant growth-promoting attributes. ZSB play a role in the solubilization of insoluble forms of Zn compounds in soil and are therefore considered “natural biofortifying agents.” The process of Zn solubilization occurs via organic acids and other chelating agents (Hussain et al., 2018). Organic acids produced by ZSB enhance soil Zn accessibility through the sequestration of cations and a reduction in rhizospheric pH (Mumtaz et al., 2017; Upadhayay et al., 2018). ZSB must possess multiple plant growth-promoting traits, which should be tested in the lab before selecting the best ZSB strain. Various studies have illustrated the multiple plant growth characteristics of ZSB, such as the production of iron-chelating compounds, i.e., “siderophores” (Costerousse et al., 2017; Upadhayay et al., 2022c), “ammonia” (Mumtaz et al., 2017), “EPS” (Khan et al., 2023), “HCN” (Mumtaz et al., 2017), phytohormones (IAA; Bhatt and Maheshwari, 2020; Kumar S. et al., 2019; Mumtaz et al., 2017; Singh et al., 2022), “Phytase” (Bhatt and Maheshwari, 2020), and “ACC deaminase” (Kumar S. et al., 2019; Singh et al., 2022). Another important feature is nutrient solubilization, e.g., solubilization of “phosphates (P)” (Bhatt and Maheshwari, 2019, 2020; Shaikh and Saraf, 2017) and “potassium (K)” (Gontia-Mishra et al., 2017). This is crucial because the complexed forms of P and K existing in the soil are not readily available to plants. The organic acid production behavior of ZSB can therefore address this issue and convert insoluble forms of P and K into soluble forms that can be used by plants.

**Figure 2:**
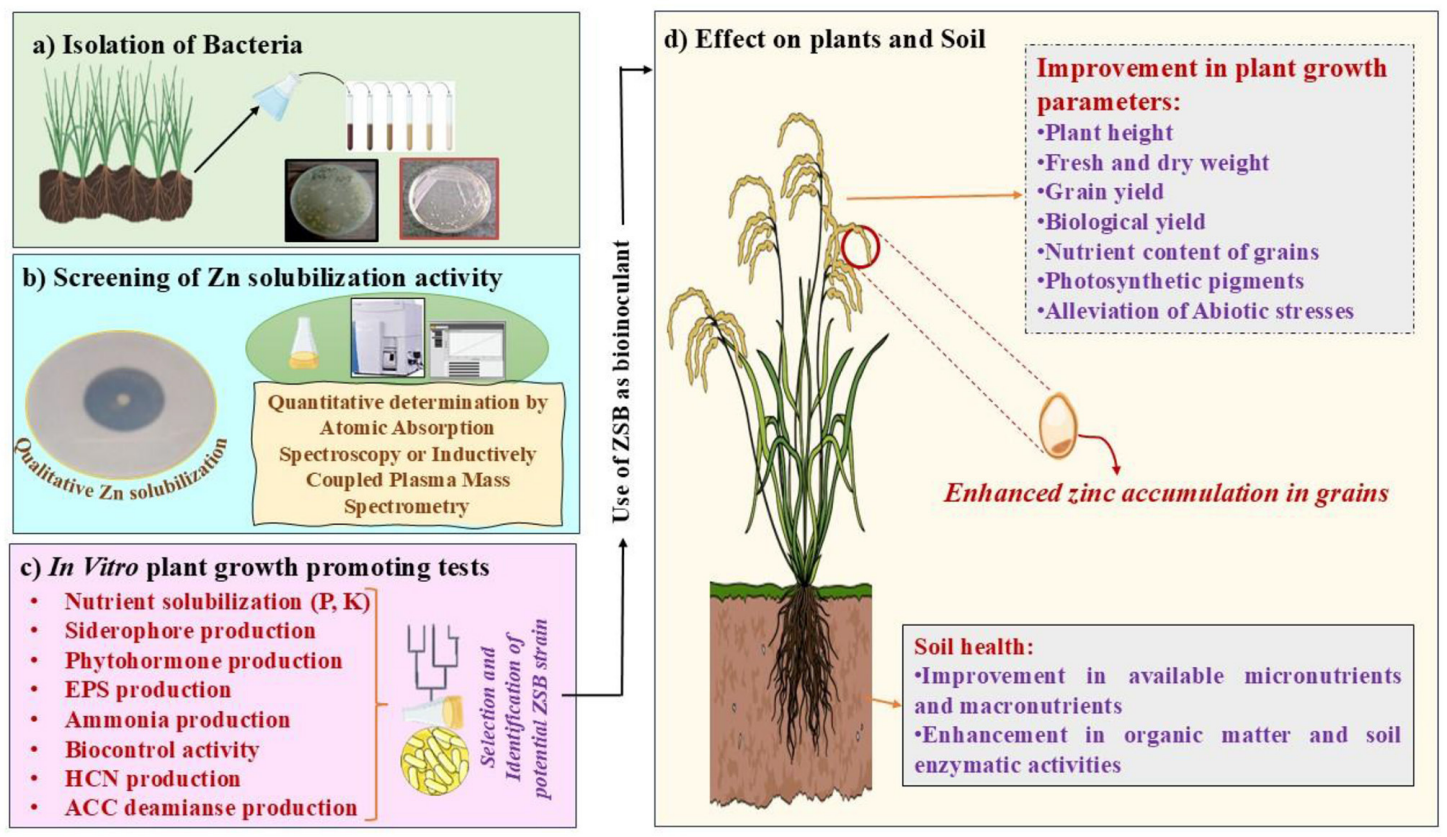
A conceptual framework showing the sequential steps involved in the isolation, screening, and functional application of potential ZSB strains for enhanced plant growth, zinc biofortification, and soil health management in sustainable agriculture. **(a)** Isolation of rhizospheric bacterial strains associated with crop plants; **(b)** Evaluation of Zn-solubilizing potential through qualitative plate assays and quantitative determination via atomic absorption spectroscopy (AAS) or inductively coupled plasma mass spectrometry (ICP-MS); **(c)** *In vitro* characterization of selected ZSB strains for various plant growth-promoting traits; **(d)** Field application of competent ZSB strains resulting in enhanced plant growth, Zn enrichment in grains (biofortification), and improved soil health.

Although the ZSB strain plays a significant role in providing a soluble form of Zn through the solubilization of complex Zn forms of Zn, its advantageous effects (e.g., increased plant growth, nutrient uptake, and yield characteristics) make it a valuable probiotic for several crops (Bhatt and Maheshwari, 2019, 2020; Upadhayay et al., 2022a). Shifting toward more eco-friendly farming practices using ZSB as bioinoculants can reduce the heavy dependency on chemical fertilizers. Therefore, promoting ZSB as bioinoculants could be an inexpensive and sustainable option for increasing Zn micronutrient levels in plants. Several bacteria have been studied for their positive attributes in Zn solubilization and for delivering appropriate amounts of Zn to crops to provide biofortification benefits (Upadhayay et al., 2022a,b; Khan et al., 2023; Rahman et al., 2024). ZSB have demonstrated growth-promoting effects in plants, such as increases in shoot and root length, fresh and dry weight, crude protein, fiber, gluten, and minerals (Hussain et al., 2020; Shaikh and Saraf, 2017). Two strains of *Bacillus aryabhattai* (“MDSR7” and “MDSR14”) enhanced Zn uptake in wheat and soybeans grown in Zn-deficient soils (Ramesh et al., 2014). “*Exiguobacterium auranticum*” has been identified as an economical option for biofortification, as it increased zinc uptake (18.2 ppm) in wheat grains (Shaikh and Saraf, 2017). Kamran et al. (2017) demonstrated that *Pantoea agglomerans* and *Enterobacter cloacae* increased Zn content in wheat shoots by 17.85 and 18.25 mg/kg, respectively, exhibiting significant potential for Zn biofortification. Moreover, in the same study, *Pantoea agglomerans* exhibited the highest Zn level (42.96 mg/kg) in the root portion. Tariq et al. (2007) showed the efficient role of inoculants in alleviating symptoms associated with Zn deficiency and their role in enhancing Zn levels in paddy grains. Moreover, the same study highlighted the important role of ZSB in improving various growth- and productivity-related traits of rice plants, such as increased biomass and grain yield. In addition to bacteria, mycorrhizal fungi have also been found to be beneficial in micronutrient biofortification (Upadhayay et al., 2019). The association of mycorrhizal fungi also increases the Zn and other micronutrient concentrations in the edible parts of crops. For instance, inoculation with locally sourced arbuscular mycorrhizal fungi (AMF) significantly improved sorghum grain nutritional quality on Sudan's vertisols, elevating bioavailable Zn concentration (40.3%), while reducing phytate content (an antinutritional factor; Elsafy et al., 2025). Two cold-adaptive bacterial strains, “*Pseudomonas jesenii* (MP1)” and “*P. palleroniana* (N26),” exhibited substantial increases in protein and Zn content in kidney beans (Khan et al., 2023). Some important ZSB that provided Zn biofortification in crop plants are shown in Table 2. Figure 2 illustrates the sequential approach for selecting potential ZSB strains and their beneficial effects on plant growth, Zn enrichment of grain, and soil health.

**Table 2:** Effect of zinc-solubilizing bacterial strains on zinc enrichment in various crops.

**ZSB**	**Plant**	**Plant part**	**Zinc concentration (mg/kg or % increase)**	**References**
*Pseudomonas protegens*	Wheat	Grain	29.33 mg/kg	Singh et al., 2022
Indigenous beneficial microbes	Wheat	Grain	141% increase (irrigated), 130% increase (rainfed)	Ali et al., 2024
*Bacillus megaterium*	Capsicum annuum	Fruit	0.25 mg/100 g	Bhatt and Maheshwari, 2020
*B*. *megaterium* CHW-22	Wheat	Grain	46.44 μg/g	Yadav et al., 2023
*Serratia marcescens* FA-4	Rice	Grain	21.4–27.7 mg/kg (pot), 18.7–30.1 mg/kg (field)	Shakeel et al., 2023
*Pseudomonas jesenii* MP1	Kidney bean	Seed	53.66%	Khan et al., 2023
*Pseudomonas kilonensis* (CDS7)	Tomato	Fruit	3.13 mg/100 g	Karnwal, 2023
*Serratia marcescens* SCHN1	Wheat	Grain	80 mg/kg	Kukreti and Singh, 2024
*Burkholderia cepacia* (BMRR126) + ZnO	Rice	Grain	33.25 mg/kg	Upadhayay et al., 2022c
Isolate HRM29	Wheat	Grain	44.54 mg/kg	Khan S. T. et al., 2022

## 5 Mechanisms of zinc solubilization

The solubilization of Zn is a prominent factor for microbial-assisted Zn biofortification. *In vitro* studies are first performed to characterize the solubilizing potential of insoluble Zn compounds by bacterial isolates. The evaluation of Zn solubilization of the putative bacterial isolates is conducted through qualitative and quantitative means (Figure 2). The formation of a halo zone on zinc minimal media around the bacterial colony provides a qualitative method for assessing the Zn solubilization potential of bacterial strains. For quantitative evaluation, atomic absorption spectroscopy (AAS), FE-SEM-EDS, and ICP-MS are primarily employed to further determine the Zn-solubilizing efficacy of the test bacterial isolate in broth containing the ingredients of minimal media and insoluble Zn compounds (Costerousse et al., 2017; Upadhayay et al., 2022c; Choudhary et al., 2024). Most of the *in vitro* studies have been accomplished in this manner. The formation of a halo zone around the bacterial colony on the minimal media plate amended with an insoluble zinc source demonstrates the pattern of Zn solubilization through the secretion of organic acids as a primary screening method under *in vitro* conditions. The possible mechanisms of Zn solubilization by bacteria can be outlined as follows: (a) acidification (through proton extrusion), (b) chelation (via production of organic acids or siderophores), and (c) chemical transformation (e.g., involving redox reactions, usually under extreme conditions; Costerousse et al., 2017; Upadhayay et al., 2022a; Nosheen et al., 2021; Jalal et al., 2024; Sethi et al., 2025). Protons can be released from bacterial cells in two ways (direct and indirect). The first involves direct release from bacterial cells into the external medium via various membrane-associated pumps, which establish ionic gradients essential for nutrient acquisition (Fasim et al., 2002). The second entails indirect release from the carboxylic groups of released organic acids (Costerousse et al., 2017). Protons can replace Zn cations at mineral sorption sites (e.g., oxides and phosphates), which in turn mobilizes Zn in solution (Glasauer et al., 2013). The chelation of metal by organic acids is typically regulated by pH. The fully deprotonated anionic forms of organic acids are usually found in neutral to alkaline environments. In this state, they can effectively function as metal-complexing agents (Costerousse et al., 2017). The involvement of “gluconic acid” and “2-ketogluconic acid” in culture broth was attributed to Zn solubilization (Shaikh and Saraf, 2017; Srithaworn et al., 2023). Costerousse et al. (2017) demonstrated that “glucose” in the culture broth promoted the production of organic acids, such as gluconic acid, malonic acid, oxalic acid, by ZSB, which eventually led to the acidification of the broth and solubilization of Zn. The solubilization of zinc oxide (ZnO) by ZSB in liquid culture was mediated through the acidification of the medium via the secretion of organic acids (lactic, acetic, succinic, and formic acids), as shown by Mumtaz et al. (2019). Glucose metabolism via oxidative phosphorylation, coupled with the activity of PQQ-dependent glucose- and gluconate-dehydrogenases, likely enables the production of gluconic acid and its derivatives in the medium (Mumtaz et al., 2019). Some arbuscular mycorrhizal (AM) fungi also dissolved “Zn phosphate” by producing organic acids in the rhizospheric region (Martino et al., 2003), and a decrease in the pH of the rhizospheric soil when inoculated with AM fungi aided in the release of Zn from the “mineral fraction” (Upadhayay et al., 2019). In general, a decrease in soil pH is key to releasing numerous nutrients (micro and macronutrients) in the rhizosphere. Lowering the pH of the soil by one unit can enhance the accessibility of Zn a 100-fold (Khan S. T. et al., 2022). However, the extent of the decrease in soil pH depends on various factors (such as soil type, soil texture, geographic location, and associated microbial communities in the soil). The increased reactivity and low persistence of Zn in the soil lead to the formation of Zn metal complexes. This event hinders the availability of Zn to plants. Naturally occurring and synthetic Zn-chelating compounds also demonstrate efficacy in improving the bioavailability of Zn in the rhizosphere (Obrador et al., 2003). Chelation is another crucial mechanism employed by plant root systems and bacteria in the rhizospheric milieu to enhance Zn bioavailability in soil. The secretion of chelating compounds and bacterial metabolites (and siderophores) binds with Zn and forms a complex, thus reducing the interaction of Zn^2^? within the soil. These Zn complexes release Zn^2+^ close to the root for absorption, allowing chelators to interact with additional Zn^2+^ (Haroon et al., 2022). Siderophores, however, assist in chelating mineral ions, primarily Fe, but are also found to chelate Zn^2+^ (Hussain et al., 2018; Verma et al., 2021; Singh D. et al., 2024; Sethi et al., 2025).

## 6 Microbial consortia as “splendid plant probiotic agents”

Chemical fertilizer application is associated with several drawbacks, such as harmful effects on the environment, soil acidification, soil fertility deterioration, water eutrophication, air pollution, and other issues (Gouda and Saranga, 2018; Menéndez and Paço, 2020). Thus, it is important to use alternative strategies, such as “plant growth-promoting bacteria (PGPB)” as potential “plant probiotics (PPs)” (Jiménez-Gómez et al., 2018; Menéndez and Paço, 2020). Using PGPB can significantly reduce dependency on chemical fertilizers (Soumya et al., 2020). The use of PPs has shown promising outcomes in improving traits linked to growth and yield attributes (Rahman et al., 2018) and plant quality (Jiménez-Gómez et al., 2018). PPs can be utilized as bioinoculants for producing functional plant-based foods with improved bioactive compounds. For instance, in addition to enhancing strawberry plant quality and yield, *Phyllobacterium* noticeably increased the amount of vitamin C (Flores-Félix et al., 2015). However, the use of two or more PGPB as a “consortium” for improved plant growth and yields is becoming more popular (Menéndez and Paço, 2020; Chaudhary et al., 2023). Competent bioinoculants based on microbial consortia can efficiently colonize the rhizosphere, improve nutrient accessibility, and increase stress tolerance in plants against various abiotic stressors (Santoyo et al., 2021). In particular, PGPR consortia often show a more significant impact on plant growth attributes (such as enhanced biomass accumulation and productivity) than single inoculants (Backer et al., 2018; Oluwambe and Kofoworola, 2016; Liu et al., 2023). Mixed rhizospheric and endophytic inoculants with extensive plant probiotic traits enhance plant biomass and improve the assimilation pattern of various essential micronutrients by plants (Emami et al., 2018). Consortia based on PPs also possess effective disease-suppressive properties and thus exert minimal negative effects on the plant (Zhang et al., 2020). The superior performance of consortia is due to the synergistic effects of different bacteria providing nutrients, eliminating inhibitory products, and benefiting each other through physical or biochemical traits (Barea et al., 2002; Molina-Romero et al., 2017). Improved resilience in plants against abiotic stressors (such as drought, salinity, and heavy metal toxicity) via PGPR-based consortia is well-documented, demonstrating modulation in physiological and biochemical pathways (Sharma et al., 2023). Moreover, their application may reduce reliance on agrochemicals and provide a greener approach to maintaining optimum crop productivity. Shahzad et al. (2014) showed that the inoculation of chickpeas with a consortium of *S. marcescens*—SF, *Serratia* sp.—ST9, and *M. cicero* increased the number and dry mass of nodules, number of pods, grain yield, chlorophyll, and protein content under both irrigation and rainy conditions. A bacterial consortium consisting of *Pseudomonas* sp., *Azotobacter chroococcum*, and *Priestia megaterium* potentially improved plant growth, grain yield, and soil nutrient status in *Cajanus cajan* (Priyanka et al., 2025). Using spontaneous antibiotic-resistant mutants, the persistence of these strains was successfully tracked across developmental stages, demonstrating their contribution to improving rhizospheric microbial abundance and P cycle gene expression, emphasizing their potential as sustainable bioformulations for crop productivity. Furthermore, Table 3 shows the agro-advantageous effects of various microbial consortia, indicating their positive impacts on plant growth, stress tolerance, nutrient uptake, and overall crop productivity.

**Table 3:** Plant growth stimulatory effects of microbial consortia across diverse crops.

**Microbial consortium designation**	**Host plant**	**Plant growth stimulatory benefits**	**References**
*Bacillus megaterium* + *Arthrobacter chlorophenolicus* + *Enterobacter* sp. + *Pseudomonas aeruginosa*	Wheat	Increase in plant height, grain yield, straw yield, higher test weight, and improvement in nutrient acquisition	Kumar et al., 2021
*Bacillus* sp. + *Delftia* sp. + *Enterobacter* sp. + *Achromobacter* sp.	Tomato (*Solanum lycopersicum* L.)	Improvement in salt tolerance, dry weight of leaf, shoot, root, shoot length, root length, chlorophyll content, and nutrient uptake	Kapadia et al., 2021
PGPR consortium (*B. megaterium* + *Paenibacillus polymyxa* + *Bacillus* sp.)	Cotton (*Gossypium hirsutum* L.)	Increase in antioxidant activities; enhancement in growth (root length, shoot length, root biomass, shoot biomass, improved yield (single boll weight and lint percentage), reduced sooty mold disease incidence)	Luqman et al., 2025
*Enterobacter ludwigii* + *Micrococcus indicus* + *Pseudomonas gessardii*	Eggplant (*Solanum melongena* L.)	Increase in root/shoot length and biomass, enhanced chlorophyll, carotenoids, total soluble sugar, and phenolic content	Kaur et al., 2024
*Trichoderma afroharzianum* + *Pseudomonas fluorescens* + *Bacillus licheniformis* + *B. subtilis*	Cluster Bean (*Cyamopsis tetragonoloba*)	Disease control against *Rhizoctonia bataticola*, increase in systemic resistance, improved plant growth, fresh/dry weight, number of pods, and seed yield	Singh S. et al., 2024
*Bacillus cereus* + *B. thuringiensis* + *Herbaspirillum huttiense*	Wheat (*Triticum aestivum*)	Reduction in Pb/Cd accumulation in shoots, improvement in root development, soil enzyme activity	Zhu et al., 2024
*Erwinia* sp. EU-B2SNL1 (N-fixer) + *C. arthrosphaerae* EU-LWNA-37 (P-solubilizer) + *P. gessardii* EU-MRK-19 (K-solubilizer)	Barley (*Hordeum vulgare*)	Enhancement in root/shoot length, biomass, chlorophyll, carotenoids, phenolics, flavonoids, and soluble sugar content	Kaur et al., 2022
*Bacillus* sp. + *Acinetobacter* sp. (Halotolerant consortium)	Maize (*Zea mays*)	Enhancement in shoot and grain N, straw and grain P. Increase in plant height, grain yield, cob length/weight, stomatal conductance, water-use efficiency, and photosynthetic rate under salinity stress	Shabaan et al., 2022
*Ochrobactrum anthropic* + *Pseudomonas palleroniana* + *P. fluorescens* + *P. palleroniana*	Rice–wheat	Enhancement in macronutrient (N, P, K, Ca, Na) uptake; Increase in 1,000 grain weight, grains per panicle/spike, tillers. grain and straw yield	Chandra and Sharma, 2021
Consortium 1 and consortium 2 (PGPR-based)	Barley (drought-sensitive and -tolerant cultivars)	Enhancement in drought tolerance, improvement in relative water content, reduction in electrolyte leakage, increase in proline, total soluble sugars, catalase, and ascorbate peroxidase activities; enhancement in root and vegetative shoot dry weight, improvement in grain yield and thousand-grain weight	Ferioun et al., 2024

## 7 Need for zinc-solubilizing bacterial consortium

Agriculturally important microbes help plants achieve optimal growth through different mechanisms, such as improving nutrient uptake, promoting root and shoot development, and enhancing soil health. They also play a significant role in mitigating the adverse effects of various abiotic stresses on plants. Microbes employ a range of tactics to help plants survive under challenging environmental conditions, including the production of stress-relieving compounds, modulation of plant metabolism, and an increase in water and nutrient use efficiency (Upadhayay et al., 2023; Yuan et al., 2024). However, microbial performance in field conditions often does not match the success observed in controlled settings. This discrepancy is attributed to variations in factors such as low bioinoculant viability, competition with native microorganisms, or the inability of single microbial strains to acclimatize to the natural environment (Ayala-Zepeda et al., 2024). Therefore, microbial consortia that combine multiple microbial strains possessing various beneficial properties provide a more effective solution for sustainable agriculture as a greener approach. Microbial strains in a consortium offer synergistic benefits for plant growth and stress resilience (Adeleke et al., 2024). As a key component of agriculturally important microbes, zinc-solubilizing bacteria (ZSB) have garnered substantial attention for their role in enhancing Zn bioavailability for plants. Although Zn is crucial for numerous plant physiological functions, its scarcity in soil can negatively impact crops by reducing both yield and quality. In the soil, ZSB contribute by solubilizing Zn from its insoluble forms through mechanisms such as organic acid production, proton extrusion, and chelation (Upadhayay et al., 2022a). Thus, the soluble form of Zn becomes readily accessible to the plant (Bhatt and Maheshwari, 2020; Mumtaz et al., 2020). Consequently, ZSB inoculants can promote the biofortification of crops in regions where Zn-deficient soils are prevalent. This approach is essential for addressing micronutrient deficiencies and supporting global food security. Although increasing the Zn content of food crops is the primary objective of employing ZSB, it is a low-cost strategy that, in addition to improving crop productivity, can also enhance farmer profitability (Upadhayay et al., 2022b). As effective biostimulants, ZSB offer numerous agronomic benefits, including (a) reducing the application of chemical fertilizers, (b) improving crop yield and overall crop quality, (c) restoring the natural equilibrium of agroecosystems, (d) enhancing the nutritional status of crops, and (e) strengthening plant resilience against abiotic stressors (drought, salinity, etc.; du Jardin, 2015; Woo and Pepe, 2018). While there is a wide array of studies demonstrating promising results from using individual ZSB strains in Zn biofortification across various crops, studies showcasing the contributions of ZSB-based consortia in Zn biofortification remain limited. The incorporation of ZSB consortia holds great potential for advancing microbial-assisted biofortification programs, providing more efficient and eco-friendly solutions for agriculture. The development of ZSB-based consortia, through strategic selection and integration of diverse ZSB strains exhibiting complementary functional attributes, has the potential to realize the benefits of biofortification, improve plant stress tolerance, and facilitate better nutrient uptake by plants (Menéndez and Paço, 2020). The development of bacterial consortia is a pivotal research area in agricultural microbiology with far-reaching implications for enhancing crop resilience, productivity, and soil health. The development of ZSB-based consortia may deliver key benefits:

➢ Using a ZSB consortium may provide more Zn biofortification benefits compared to a single ZSB strain (it acts as an auxiliary factor residing in the soil, supplying soluble fractions of Zn to plants and ultimately alleviating Zn deficiency in plants). Its application may curb the rampant application of agrochemicals.
➢ The consortium can include more than one strain with various plant growth-promoting traits, such as nitrogen fixation, phosphate solubilization, and plant growth hormone production, which are essential for enhancing crop performance.
➢ Acting as effective plant probiotics, the ZSB-based consortium can stimulate root development, improve root and shoot length and biomass, enhance nutrient absorption, and increase crop yield.
➢ These consortia improve plant stress resilience and have a positive impact on soil health.

## 8 Role of zinc-solubilizing bacterial consortia and other bacterial consortia in zinc biofortification

Bacterial consortia, in addition to demonstrating multiple plant growth-promoting traits, are also recognized for their role in increased nutrient assimilation in food crops. Very few studies have investigated this approach for developing zinc-fortified crops using bacterial consortia. However, a substantial body of literature exists detailing the role of specific bacterial strains in Zn biofortification rather than bacterial consortia. Limited research has shown the potential of microbial consortia in micronutrient biofortification. The rhizobacterial consortium (one strain of *Burkholderia* sp. and two strains of *Acinetobacter* sp.) enhanced the residual effect of applied zinc and improved Zn uptake in the grain and straw of wheat (Vaid et al., 2019). Rezaeiniko et al. (2019) demonstrated the contribution of a consortium (*Enterobacter cloacae* and *Bacillus megaterium*) combined with Zn-sulfate fertilizer. The results were evident in the highest levels of soil exchangeable Zn, increased Zn uptake in grain, and improved grain yield. The combination of plant growth-promoting bacteria CP4 (*Bacillus subtilis*) and AM fungi yielded better results in terms of increased micronutrient and macronutrient concentrations in wheat grains. In addition to showing nutritional benefits, consortium application also improved several yield-associated parameters (thousand-grain weight, number of tillers per plant, and grains per spike; Yadav et al., 2020). Improved Zn accumulation in plant shoots was observed when a consortium comprising *Pseudomonas* sp. and *R. leguminosarum* was used as a bioinoculant (Mishra et al., 2012). A ZSB consortium of “*Bacillus* sp. (SH-10)” and “*B. cereus* (SH-17)” served as a competent biofortifying agent, producing Zn-enriched rice grains and demonstrating a maximum Zn translocation index (1.6–1.7) compared to other treatments (Shakeel et al., 2015). The use of Zn-biofertilizer developed from a consortium of two ZSB strains, namely Streptomyces sp. and *Pseudomonas* sp., along with ZnO nanoparticles, resulted in Zn biofortification by significantly increasing Zn levels in wheat grains (43.0 mg/kg; Saleem and Khan, 2025). Additionally, feeding Wistar rats flour derived from the biofortified grains resulted in elevated blood plasma Zn levels (7.79 μg/mL), indicating effective Zn bioavailability from plant sources to animals. A consortium developed using Burkholderia and Acinetobacter achieved maximum Zn accumulation in rice grains of two cultivars, “PD16” (16.1 mg/kg) and “NDR359” (16.0 mg/kg; Vaid et al., 2014). Kumar et al. (2017) assessed the effectiveness of a consortium (*Enterobacter* and *S. marcescens*) on Zn content in wheat, resulting in a 32% increase in the pot trial and a 23% increase in the field trial. Furthermore, the consortium also enhanced the concentration of other micronutrients, with increases in Cu (56%), Mn (52%), and Fe (18%) in pot trials, and Cu (43%), Mn (48%), and Fe (16%) in field conditions. A consortium developed from two *Pseudomonas* species, *P. jessenii* and *P. synxantha*, showed a significant effect on Zn accumulation in rice seeds compared to the control (without bacterial inoculation; Gusain and Sharma, 2019). The highest Zn content in the grain, 25.07 mg/kg, was noted for the treatment consisting of a consortium of ZSB BMRR126 (*B. cepacia*) and BMAR64 (*Pantoea rodasii*) along with zinc oxide (ZnO) in the Terai region (Upadhayay et al., 2022c). The triple combination of bacterial strains (*B. megaterium, A. chlorophenolicus*, and _*Enterobacter* sp.) significantly improved the content of Zn, Cu, Mn, and Fe by 58.5%, 83.0%, 104.0%, and 49.2%, respectively, in pot trials and by 62.8%, 98.6%, 95.0%, and 42.4%, respectively, in field trials (Kumar et al., 2014). Tariq et al. (2007) utilized a microbial consortium (developed from *Pseudomonas* sp. and other PGPR) that served as an effective Zn-solubilizing bioinoculant. This consortium significantly increased Zn accumulation in rice grains by up to 157% compared to untreated controls. Microbial inoculation (*Anabaena*–*Azotobacter* biofilm) demonstrated notable effects on Zn accumulation, with concentrations reaching 107.01 μg g?1 in the flag leaf, indicating a cyanobacteria-mediated process facilitating Zn uptake in maize (Prasanna et al., 2015). Jalal et al. (2021) showed that co-inoculation of *R. tropici* + *B. subtilis* with soil Zn application significantly enhanced zinc accumulation in common bean grains (54.5 mg/kg in the 2019 crop season and 60.7 mg/kg in the 2020 crop season). Sarkar et al. (2022) reported that co-inoculation of *Pseudomonas fluorescens* and *Bacillus subtilis* with 75% RDF resulted in the highest Zn uptake (65.9 g/ha) in red cabbage. The study by Singh et al. (2025) revealed that ZSB “Consortium1 (T4)” and “Consortium2 (T5)” significantly enhanced Zn bioavailability in rice varieties PD 26 and NDR 359. These treatments also improved carbonic anhydrase (CA) and superoxide dismutase (SOD) activities, along with enhanced gaseous exchange parameters and grain yield. A consortium of indigenous ZSB (including *Klebsiella* sp., *Brevibacterium* sp., *Citrobacter* sp., *Exiguobacterium* sp., *Raoultella* sp., and *Acinetobacter* sp.) improved wheat yield and Zn uptake under both irrigated and rainfed conditions. When co-applied with Zn-based fertilizer, the consortium significantly increased yield (up to 43.5%) and Zn uptake (up to 166%; Ali et al., 2023b). A consortium comprising “*Anabaena* sp. (CR1)” and “*Providencia* sp. (PR3),” along with 75% RDF and Zn, increased Zn uptake (323.8 g/h) in wheat, and this biofilmed formulation was regarded as one of the finest resources in nutrient management for wheat (Shahane et al., 2017). Table 4 provides an overview of Zn biofortification performance and associated growth enhancements driven by various microbial consortia in different crops.

**Table 4:** Zinc biofortification benefits and plant growth enhancement by microbial consortia in various crops.

**Consortium designation**	**Host plant**	**Plant part**	**Zinc concentration (mg/kg or % increase)**	**Plant growth benefits**	**References**
*Pseudomonas* spp. (VBZ4 and VBZ17)	Okra	Fruit	2.85 mg/100 g	Increase in length of shoot and root, dry weight of shoot and root, number of branches, stem girth, and number of fruits	Karnwal, 2021
*Paenibacillus polymyxa* ZM27 + *Bacillus subtilis* ZM63 + *Bacillus aryabhattai* S10	Maize	Grain	11%	Increase in plant height, fresh and dry weight of shoot and root, and grain yield	Ahmad M. et al., 2023
*Pantoea agglomerans* + *Pseudomonas fragi*	French bean	Pods	30.04 mg/kg	Enhancement in root development, biomass of roots and shoots; improvement in chlorophyll and carotenoid content; higher antioxidative enzyme activity, osmoprotectant content, and improvement in salinity stress tolerance	Gupta et al., 2023
*Cold-tolerant Enterobacter hormaechei CHM16*, and *Pantoea agglomerans HRM 23*	Kidney bean	Seeds	363.22 ppm	Increase in plant height, pod length, and No. of grain/pod, 1,000 grain weight and grain yield	Khan et al., 2024
Rhizobacterial consortium	Rice	Grain	12.0–17.0 Zn mg/kg	Increase in number of effective tillers, grain yields	Vaid et al., 2020
*B. diazoefficiens, Bacillus* sp. MN54, and *P. indica*	Soybean (*Glycine max* L.)	Grain	11.11%	Increase in plant height, number of nodules, number of pods, and grain yield Improvement in chlorophyll content, leghemoglobin contents, crude fiber, protein, and oil content	Rafique et al., 2025
Microbial consortia (Pusa decomposer) + Paddy Straw Incorporation PSI + Urea @10 kg/ha	Wheat (*Triticum aestivum* cv. HD 2967)	Grain	38.08–39.03 mg/kg	–	Manu et al., 2024
Consortium (*K. pneumoniae* Zn2 + *K. quasipneumoniae* CmA9 + *P. rettgeri* DrSrA1 + *A. vinelandii* 1CM + *B. subtilis* P18.3) + 75% NPK + organic fertilizer	Sorghum	Grain	18.48%	–	Kasno et al., 2024
*Lysinibacillus* sp. (strain VITKC-5) and *Acinetobacter* sp. (strain VITKC_6)	Tomato	Fruit	0.089 mg/fruit	Increase in the average fresh weight, superior characteristics in fruit dry weight, fruit width and fruit height elevated expression of nutrient transporter genes	Arakkal Thaiparambil and Radhakrishnan, 2023
*Bacillus aryabhattai* ZM31 + *B. subtilis* ZM63	Maize (*Zea mays*)	Grain	23%	Increase in maize yield	Mumtaz et al., 2020
*Pseudomonas* sp. (B1 & B2)	Wheat (PBW 373)	Grain	31%	Increase in plant height, chlorophyll, and grain number	Joshi et al., 2013
ZnONPs (Soil and foliar application) + ZSB consortium (*Pseudomonas aeruginosa*-YZn1 + *Stenotrophomonas maltophilia*-WZn1)	Wheat	Grain	89.06% increase over control	Significant increase in chlorophyll SPAD value, 1,000-grain weight, grain yield, harvest index, and grain Zn content	Mahmood et al., 2024
*Pseudomonas kilonensis* (CDS7) and *Pseudomonas chlororaphis* (CDS21)	Tomato (*Solanum lycopersicum*)	Fruit	2.36 mg/100 g	Significant increase in length, dry and fresh weight of shoot and root, stem girth and fruit yield	Karnwal, 2023
*Bacillus* sp. SH-10 + *B. cereus* SH-17 + *S. marcescens* FA-4	Rice (basmati 385)	Grain	29.2 mg/kg	Improvement in plant height, chlorophyll content, Zn-requiring enzymes, and grain yield	Shakeel et al., 2024
	Rice (super basmati)	Grain	30.5 mg/kg		

## 9 Effect of ZSB inoculants on soil

The soil is a suitable and dynamic hub for diverse flora and fauna and supports microbial activity. The “Rhizosphere” is the narrow zone of soil directly influenced by plant roots. It is a belowground interface characterized by complex interactions among soil, root systems, and diverse microbial communities. This region has a significant impact on nutrient cycling because of its high microbial activity (Upadhayay et al., 2023). Moreover, in this region, microbes regulate soil nutrient equilibrium by fixing nitrogen, mineralizing organic matter, and solubilizing inorganic minerals (P, K, Zn, etc.). This ensures that NPK is available at adequate levels for both plants and microbes (Kaviya et al., 2019; Upadhayay et al., 2023). Despite the abundance of Zn in the soil, crops suffer from its deficiency due to its unavailable form. Although Zn fertilizers such as Zn sulfate or Zn-EDTA are commonly used to address Zn deficiency (Ali et al., 2023a), they often prove ineffective in the long term as 96–99% of the applied Zn rapidly transforms into unavailable forms through precipitation with carbonates, oxides, or phosphates (Zhang et al., 2017; Ali et al., 2023a). ZSB plays a key role in solubilizing these insoluble Zn reservoirs. The solubilization process may be achieved through mechanisms such as the production of organic acids, proton extrusion, or the production of chelating agents by ZSB (Bhatt and Maheshwari, 2020). Organic acids increase the availability of Zn in soil by sequestration of cations and by lowering rhizospheric pH (Ali et al., 2023b). ZSB strains or their consortia, whether alone or in combination with low-cost zinc sources like ZnO, can be used in a sustainable and cost-effective strategy for improving Zn bioavailability and supporting plant growth (Saravanan et al., 2004; Upadhayay et al., 2022c). ZSB inoculants influence soil quality by improving soil nutrient status and enzymatic activities. The increased soil enzymatic activities, like dehydrogenase, urease, alkaline phosphatases, and acid phosphatases, followed by ZSB and PGPR microbial inoculation, reflect their function in maintaining soil health (Hussain et al., 2015; Singh et al., 2018, 2017; Upadhayay et al., 2021). These enzymatic activities are especially significant for soil health because of their importance in maintaining soil fertility and responding quickly to environmental changes (Li et al., 2021). Chickpea seed inoculation with ZnSB13 (*Bacillus cereus*) resulted in increased activity of rhizospheric phosphatase and dehydrogenase, thereby maximizing Zn availability in soil (Batool et al., 2021). Endophytic ZSB strains (“SaBA1,” “SaPS2,” “SaEN1,” and “SaPA1”) enhanced soil available Zn by 0.58–0.92 mg/kg, and supported tomato growth under Zn-deficient conditions (Liao et al., 2024). Inoculation of *P. protegens* (CP17) showed improvements in zinc availability (0.978 and 1.32 mg/kg) and enzymatic activities (dehydrogenase and phosphatase), facilitating Zn uptake from rhizospheric soil to wheat grain and straw under both saline and non-saline conditions (Singh et al., 2022). Inoculation with *Bacillus* (Zn-P-1) improved soil-available P and Zn, enhanced microbial biomass carbon, and reduced soil pH, facilitating improved Zn assimilation in wheat (Gupta et al., 2024). In addition to exhibiting Zn biofortification benefits in wheat, the application of *B. megaterium* (CHW-22) significantly enhanced the availability of key macronutrients in soil such as nitrogen, phosphorus, and potassium (Yadav et al., 2023). It also improved concentrations of micronutrients such as Fe (4.56 μg/g), Zn (0.92 μg/g), Cu (1.86 μg/g), and Mn (5.46 μg/g), and increased soil biological properties (DHA, APA, FDA, and SMBC). In two Central Indian Himalayan regions (Harsil and Chakrata), two bacterial strains, namely *P. jesenii* and *P*. *palleroniana*, used as potential inoculants, improved the yield attributes of kidney beans and improved soil health status (Khan et al., 2023). *P. jesenii* at Harsil improved both macro and micronutrient availability, while *P. palleroniana* at Chakrata improved N and K content, with *P. jesenii* again demonstrating the maximum micronutrient content in the soil. Moreover, *P. palleroniana* treatment exhibited improved soil enzyme activity at both locations. The ZSB consortium (BMRR126 + BMAR64) with ZnO consistently improved soil health in two different regions, i.e., *Terai and Katchar*, displaying maximum dehydrogenase activity, increased DTPA-extractable Zn, and enhanced NPK levels (Upadhayay et al., 2022c). The mixed ZSB inoculation (*A. nosocomialis* SR R-10 and *A. seifertii* SR R-12) significantly enhanced soil Zn bioavailability (6.17 mg/kg) and moderately acidified the soil (pH 5.8; Ramly et al., 2024). The mineral-solubilizing bacterial strain *Pseudomonas aeruginosa* (liquid formulation) combined with farmyard manure notably increased Zn concentration (7.24 mg/kg) and P content (49.65 kg/ha) in the soil after 120 days, and also improved the mineral content (Zn and P) of groundnut (Sunitha Kumari et al., 2023). Singh et al. (2023) explored the potential of native ZSB to augment Zn nutrition in rice grown in sodic soils, where Zn availability was inadequate due to the low solubility of Zn. The results of their study exhibited that *B. paramycoides* strain-1 significantly improved Zn uptake by rice (by 17%) and also enhanced the water-soluble and exchangeable Zn fractions in deficient soils (by 22%−24%). The combination of *Bacillus subtilis* IA6 and *Bacillus* sp. IA16 enhanced soil Zn content, exhibiting a 12.3% increase over control, and outperformed both conventional NPK fertilization and other microbial combinations (Ahmad I. et al., 2023). Moreover, in the Chakrata region, the consortium treatment (encompassing cold-tolerant bacteria) significantly enhanced soil health (kidney bean growing soil), increasing organic C content (1.62%), available NPK, available Zn content (1.2 ppm), and Fe content (36.9 ppm), along with elevated enzymatic activities (such as dehydrogenase, urease, and fluorescein diacetate hydrolysis; Khan et al., 2024). The consortium also functioned as a precision microbiome modulator, selectively amplifying *Bacillus* (23% dominance) while preserving native bacterial phyla, suggesting the potential application of bioinoculants for this agroecological region.

## 10 Conclusion

Combating Zn deficiency is a major requirement for lowering the risk of ailments associated with Zn malnutrition. Several biofortification approaches (agronomic, plant breeding, transgenic approaches, and the use of microbial inoculants) are employed to improve Zn concentration in food crops. ZSB, as “potential bio inoculants,” show significant plant growth-promoting attributes. Moreover, the higher efficacy of microbial consortia compared with single bacterial inoculants highlights the potential of mixing cultures of microorganisms for effective plant growth. The consortium of ZSB exhibited Zn micronutrient enhancement in agricultural crops and thus plays a role in “ZSB-assisted biofortification.” A wide range of mechanisms (such as secretion of organic acids, H^+^, and chelating agents) shown by ZSB aids in the solubilization of insoluble Zn compounds. The use of the ZSB consortium has been illustrated as an effective and eco-friendly approach in Zn biofortification, serving as a potential bioinoculant for sustainably improving crop growth and yield.

## 11 Future prospects

The application of microorganisms in the biofortification of crops is gaining appreciation, but future research is needed to understand the diversity of ZSB and the complex mechanisms involved in Zn solubilization. It is also required to develop potential consortia of ZSB containing “bacterial strains” modified to express desirable traits (such as increased capacity for zinc solubilization along with significant plant growth-promoting attributes) through genetic engineering. Furthermore, it is essential to explore ZSB consortia and develop their formulations as biofertilizers to improve grain mineral content, enhance nutritional quality, and boost overall crop productivity.
